# Selective facial mimicry of native over foreign speakers in preverbal infants

**DOI:** 10.1016/j.jecp.2019.01.015

**Published:** 2019-07

**Authors:** Carina C.J.M. de Klerk, Chiara Bulgarelli, Antonia Hamilton, Victoria Southgate

**Affiliations:** a Department of Psychology, University of Essex, Essex CO4 3SQ, UK; b Centre for Brain and Cognitive Development, Department of Psychological Sciences, Birkbeck College, University of London, London WC1E 7HX, UK; c Department of Medical Physics and Biomedical Engineering, University College London, London WC1E 6BT, UK; d Institute of Cognitive Neuroscience, University College London, London WC1E 6BT, UK; e Department of Psychology, University of Copenhagen, DK-1017 Copenhagen, Denmark

**Keywords:** Mimicry, Infancy, Group membership, TPJ, fNIRS, EMG

## Abstract

Mimicry, the spontaneous copying of others’ behaviors, plays an important role in social affiliation, with adults selectively mimicking in-group members over out-group members. Despite infants’ early documented sensitivity to cues to group membership, previous work suggests that it is not until 4 years of age that spontaneous mimicry is modulated by group status. Here we demonstrate that mimicry is sensitive to cues to group membership at a much earlier age if the cues presented are more relevant to infants. 11-month-old infants observed videos of facial actions (e.g., mouth opening, eyebrow raising) performed by models who either spoke the infants’ native language or an unfamiliar foreign language while we measured activation of the infants’ mouth and eyebrow muscle regions using electromyography to obtain an index of mimicry. We simultaneously used functional near-infrared spectroscopy to investigate the neural mechanisms underlying differential mimicry responses. We found that infants showed greater facial mimicry of the native speaker compared to the foreign speaker and that the left temporal parietal cortex was activated more strongly during the observation of facial actions performed by the native speaker compared to the foreign speaker. Although the exact mechanisms underlying this selective mimicry response will need to be investigated in future research, these findings provide the first demonstration of the modulation of facial mimicry by cues to group status in preverbal infants and suggest that the foundations for the role that mimicry plays in facilitating social bonds seem to be present during the first year of life.

## Highlights

• 11-month-olds selectively mimicked facial actions of native over foreign speakers.
• This selective mimicry was accompanied by greater left temporal parietal cortex activation.
• Facial mimicry is influenced by cues to group membership in the first year of life.

## Introduction

It is a common feeling: While talking to a friend or colleague, you suddenly realize that you are copying her behavior or accent. This tendency to spontaneously and unconsciously copy or “mimic” others’ behaviors has been suggested to play an important role in social interactions. For example, it contributes to the development of liking and rapport between strangers and makes social interactions more smooth and enjoyable (van Baaren, Janssen, Chartrand, & Dijksterhuis, 2009). It has been suggested that mimicry can be used as a strategy for social affiliation (Wang & Hamilton, 2012), and indeed studies have demonstrated that adults increase mimicry toward people they like and in-group members, while mimicry of out-group members is inhibited (for reviews, see Chartrand and Lakin, 2013, Hess and Fischer, 2017, van Baaren et al., 2009).

Despite the important social functions that mimicry is hypothesized to serve, surprisingly little is known about the development of this phenomenon. While reports of neonatal imitation of facial actions (e.g., Meltzoff & Moore, 1983) have been subject to much criticism and doubt (e.g., Jones, 2009, Oostenbroek et al., 2016), recent studies that used a more objective measure of mimicry (i.e., electromyography [EMG]) have demonstrated that infants exhibit mimicry of emotional and nonemotional facial actions from at least 4 months of age (de Klerk et al., 2018, Isomura and Nakano, 2016) and that this early mimicry is modulated by eye contact (de Klerk et al., 2018). However, it is unknown when other social factors, such as group membership, start to modulate infants’ spontaneous mimicry behavior. In the current study, therefore, we investigated whether mimicry is modulated by cues to group membership in 11-month-olds.

Previous research suggests that infants are sensitive to signals related to group membership from an early age (Liberman, Woodward, & Kinzler, 2017). For example, 8-month-olds expect agents who look alike to act alike (Powell & Spelke, 2013), and 10- and 11-month-olds show a preference for native foreign speakers compared to foreign speakers (Kinzler, Dupoux, & Spelke, 2007) and for puppets who share their food preferences (Mahajan & Wynn, 2012). Furthermore, 10- to 14-month-olds are more likely to adopt the behaviors of native speakers, showing a greater propensity to try foods they endorse (Shutts, Kinzler, McKee, & Spelke, 2009) and to imitate their novel object-directed actions (Buttelmann et al., 2013, Howard et al., 2015). However, in these latter studies where infants selectively imitated object-directed actions of native speakers, it is difficult to determine whether infants’ imitative behaviors were predominantly driven by affiliation with the model or by the motivation to learn normative actions from members of their own linguistic group, and most likely both learning and social goals played a role (Over & Carpenter, 2012). Whereas the conscious imitation of object-directed actions is considered an important tool for social and cultural learning, the spontaneous unconscious mimicry of intransitive behaviors is often thought to serve a predominantly social function such as signaling similarity and enhancing affiliation (e.g., Lakin, Jefferis, Cheng, & Chartrand, 2003; but see also Kavanagh & Winkielman, 2016). The current study, therefore, aimed to investigate whether nonconscious mimicry is modulated by cues to group membership in infancy. Considering the importance of facial information in our day-to-day social interactions, facial mimicry may provide a particularly strong affiliative signal (Bourgeois & Hess, 2008); therefore, we specifically focused on mimicry of facial actions. Whereas the majority of previous studies on facial mimicry used emotional facial expressions as the stimuli (e.g., Isomura and Nakano, 2016, Kaiser et al., 2017), in the current study we investigated infants’ tendency to mimic nonemotional facial actions, such as mouth opening, to ensure that we were measuring motor mimicry processes without confounding them with emotional contagion (Moody & McIntosh, 2011).

Despite infants’ early sensitivity to cues to group membership, the only previous study investigating the effect of group membership on young children’s mimicry found that 4-year-olds, but not 3-year-olds, showed modulation of overt behavioral mimicry by group status (van Schaik & Hunnius, 2016). Given that our previous work has shown that facial mimicry is already flexibly modulated by gaze direction at 4 months of age (de Klerk et al., 2018), this apparent insensitivity of behavioral mimicry to group membership cues in preschoolers may seem perplexing. One explanation for this finding may be that in the study by van Schaik and Hunnius (2016), the authors used a *minimal group* paradigm in which the in- and out-groups were defined by an arbitrary marker (i.e., t-shirt color). First devised by Tajfel (Tajfel, Billig, Bundy, & Flament, 1971), this paradigm is based on adults’ tendencies to favor those who share a superficial likeness with themselves. However, as van Schaik and Hunnius (2016) pointed out, it is not obvious that such sensitivity to superficial attributes should be present during early childhood. Alternatively, it could be that coding of overt behavioral mimicry provides a less sensitive measure compared to facial mimicry as measured by EMG.

Here, we used language as a signal of group membership instead because it has been suggested that this is a particularly potent cue to social structure (Liberman et al., 2017) and previous research has shown that infants’ behavioral preferences are modulated by this cue from at least 10 months of age (Kinzler et al., 2007). In the current study, 11-month-old infants observed videos of facial actions (e.g., mouth opening, eyebrow raising) performed by models who spoke to the infants in their native language (English; Native speaker condition) or an unfamiliar foreign language (Italian; Foreign speaker condition) while we measured activation of their mouth and eyebrow muscle regions using EMG to obtain an index of mimicry. EMG captures the subtle muscle changes that occur during automatic facial mimicry and likely provides a more objective and sensitive measure of mimicry compared to that measured by observational coding. This is important for studying facial mimicry in developmental populations, especially considering the controversy surrounding facial mimicry in newborn infants (e.g., Jones, 2009, Oostenbroek et al., 2016). We simultaneously used functional near-infrared spectroscopy (fNIRS) to investigate the neural mechanisms underlying any differential mimicry responses, allowing us to potentially shed more light on the underlying cognitive mechanisms.

Given infants’ preference for linguistic in-group members (Kinzler et al., 2007, Liberman et al., 2017), previous work demonstrating that behavioral, vocal, and facial mimicry are modulated by group membership in adults (e.g., Bourgeois and Hess, 2008, Weisbuch and Ambady, 2008, Yabar et al., 2006), and previous work demonstrating that even in infancy facial mimicry can be flexibly deployed (de Klerk et al., 2018), we hypothesized that infants would show greater facial mimicry of native speakers compared to foreign speakers.

In terms of neural activation, previous functional magnetic resonance imaging (fMRI) research with adult participants has highlighted several regions that may be involved in the selective mimicry of linguistic in-group members. First, the temporal parietal junction (TPJ) has been suggested to play an important role in self–other differentiation (Uddin, Molnar-Szakacs, Zaidel, & Iacoboni, 2006) and in the control of imitative responses (Hogeveen et al., 2015, Spengler et al., 2009). Furthermore, previous work has demonstrated enhanced TPJ activation for interactions with in-group members (Rilling, Dagenais, Goldsmith, Glenn, & Pagnoni, 2008) and during mimicry in an affiliative context (Rauchbauer, Majdandžić, Hummer, Windischberger, & Lamm, 2015). It has been suggested that the TPJ may be of particular importance in situations where salient affiliative signals lead to a greater tendency to mimic, requiring a greater effort to disambiguate one’s own actions from those of others (Rauchbauer et al., 2015). Based on these findings, we hypothesized that if linguistic group status is indeed perceived as an affiliative signal leading to enhanced facial mimicry, infants would exhibit greater activation of temporoparietal regions during the observation of facial actions performed by the native speaker compared to the foreign speaker driven by the enhanced effort needed to differentiate their own actions from those of the model. The medial prefrontal cortex (mPFC) is another area that may play a role in the current study given that it has been implicated in reasoning about similar others (Mitchell, Banaji, & MacRae, 2005), shows greater activation when interacting with in-group members (Rilling et al., 2008), and has been shown to be involved in modulating mimicry (Wang and Hamilton, 2012, Wang et al., 2011). Based on these findings, we hypothesized that infants would also exhibit greater activation over prefrontal areas during the observation of facial actions performed by native speakers compared to foreign speakers.

## Method

### Participants

A total of 55 11-month-old infants observed the stimuli (for a description, see “Stimuli and procedure” section) while we simultaneously measured their facial muscle responses using EMG and their neural responses using fNIRS. The final sample consisted of 19 infants who provided sufficient data to be included in the EMG analyses (*M*_age_ = 343 days, *SD* = 15.50, range = 316–375; 4 girls) and 25 infants who provided sufficient data to be included in the fNIRS analyses (*M*_age_ = 342 days, *SD* = 14.99, range = 310–375; 9 girls). This study was part of a longitudinal project investigating the development of mimicry, and we tested all the infants who were able to come back for this second visit of the project. Power analyses using effect sizes based on the previous visit of the project, where we found evidence for social modulation of facial mimicry at 4 months of age (de Klerk et al., 2018), revealed that a total sample size of 12 participants would have provided enough power (.95 with an alpha level of .05) to identify similar effects.

Because we tried to get the infants to wear both the fNIRS headgear and the EMG stickers on their face, the dropout rate for this study was relatively high, but still comparable to other neuroimaging studies with a similar age range that used only one method (e.g., de Klerk et al., 2015, Stapel et al., 2010, van Elk et al., 2008).

A total of 36 infants were excluded from the EMG analyses due to technical error (*n* = 8), because they did not provide enough trials for analyses due to fussiness (*n* = 14) or inattentiveness (*n* = 8), or because they constantly vocalized or repeatedly put their fingers in their mouth (*n* = 4)—factors that were likely to have resulted in EMG activity unrelated to the stimulus presentation. A total of 30 infants were excluded from the fNIRS analyses due to a refusal to wear the fNIRS headgear (*n* = 6), too many bad channels (*n* = 2), or because they did not provide the minimum of 3 good trials per condition due to fussiness (*n* = 14) or inattentiveness (*n* = 6). One additional infant was excluded from both the fNIRS and EMG analyses because she was exposed to Italian at least once a week, and another infant was excluded because the older sibling had recently been diagnosed with autism spectrum disorder—a disorder with a strong genetic component (Ozonoff et al., 2011) in which spontaneous facial mimicry has been found to be atypical (McIntosh, Reichmann-Decker, Winkielman, & Wilbarger, 2006). Because we did not selectively include only monolingual infants in the original sample for the longitudinal project, 4 of the included infants were bilingual but heard English at least 60% of the time. One additional infant was bilingual Italian (heard 75% of the time) and French (heard 25% of the time), and for him the Italian speaker was coded as the native speaker.^1^ Although one might expect weaker in- and out-group effects in bilingual infants, previous research suggests that both monolingual and bilingual children prefer in-group members who use a familiar language (Souza, Byers-Heinlein, & Poulin-Dubois, 2013). All included infants were born full-term, healthy, and with normal birth weight. The study received approval from the institutional research ethics committee. Written informed consent was obtained from the infants’ caregivers.

### Stimuli and procedure

**Fig. 1:**
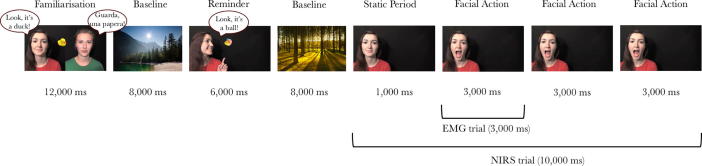
Schematic overview of the stimulus presentation. Infants first observed 2 Familiarization trials, during which the two models took turns labeling familiar objects in either English or Italian. Thereafter, Reminder trials, during which one of the models labeled a familiar object, and Facial Action trials, during which the same model performed facial actions such as mouth opening and eyebrow raising, alternated.

The experiment took place in a dimly lit and sound-attenuated room, with the infants sitting on their parent’s lap at approximately 90 cm from a 117-cm plasma screen. Infants were presented with videos of two models who spoke either English (Native speaker) or Italian (Foreign speaker). Infants first observed 2 Familiarization trials during which the models labeled familiar objects in either English or Italian. Thereafter, Reminder trials, during which one of the models labeled a familiar object, and Facial Action trials, during which the same model performed facial actions such as mouth opening and eyebrow raising, alternated (see Fig. 1). Facial Action trials started with 1000 ms during which the model did not perform any actions, followed by her performing three repeats of the same facial action, each lasting 3000 ms. The Reminder and Facial Action trials were alternated with 8000-ms Baseline trials consisting of pictures of houses, landscapes, and landscapes with animals to allow the hemodynamic response to return to baseline levels. The order of trials during the Familiarization phase was randomized, and the order of trials during the Reminder and Facial Action phases was pseudorandomized to ensure that infants saw a roughly equal number of eyebrow and mouth actions. Videos were presented until the infants had seen 12 10-s Facial Action trials (6 Native and 6 Foreign) or until the infants’ attention could no longer be attracted to the screen. Both the EMG and fNIRS analyses focused only on the Facial Action trials. The role of the models (Native vs. Foreign speaker) was counterbalanced across infants. To validate our procedure as tapping infants’ preference for native speakers, at the end of the session infants were encouraged to choose between the two models by reaching for a picture of one of the two models presented on cardboard. The cardboard with the pictures was brought into the room by an experimenter who was unaware which of the models in the videos had been the native speaker. The experimenter held the cardboard in front of the infants without saying anything to avoid biasing them toward choosing the native speaker.

### Video coding and data exclusion

Videos were coded offline to determine which trials could be included in the analyses. Note that because the EMG and fNIRS signals have very different temporal resolutions and are influenced by different types of noise, the exclusion criteria for the EMG and fNIRS trials were not identical. For example, whereas facial mimicry as measured by EMG can be recorded on a millisecond scale, the hemodynamic response takes several seconds to build up. Therefore, each 3000-ms period during which a facial action was performed by the model in the video was treated as a separate EMG trial, whereas for the fNIRS analyses we treated the 10-s videos including three repeats of the same facial action as one trial (see Fig. 1).

EMG trials during which the infants did not see at least two thirds of the action, or trials during which the infants vocalized, smiled, cried, or had something in their mouth (e.g., their hand), were excluded from the analyses because EMG activity in these cases was most likely due to the infants’ own actions. EMG trials during which the infants pulled or moved the EMG wires were also excluded, as were trials during which there was lost signal over either of the electrodes. Only infants with at least 2 trials per trial type (Native_Mouth, Native_Eyebrow, Foreign_Mouth, or Foreign_Eyebrow) and at least 6 trials per condition (Native_FacialAction vs. Foreign_FacialAction) were included in the EMG analyses (previous infant EMG research used a similar or lower minimum number of included trials; e.g., Isomura and Nakano, 2016, Turati et al., 2013). On average, infants contributed 11 EMG trials per condition to the analyses: 6 trials for the Native_Mouth trial type (range = 3–9), 5 trials for the Native_Eyebrow trial type (range = 2–8), 6 trials for the Foreign_Mouth trial type (range = 2–9), and 5 trials for the Foreign_Eyebrow trial type (range = 2–9). The number of included EMG trials did not differ between the Native and Foreign conditions (*p* = .509).

fNIRS trials during which the infants did not attend to at least two of the three facial actions, or trials during which the infants were crying, were excluded from analyses. We also excluded Baseline trials during which the infants were looking at their parents’ face or their own limbs. Only infants with at least 3 trials per experimental condition (Native_FacialAction vs. Foreign_FacialAction)^2^ were included in the fNIRS analyses (Lloyd-Fox et al., 2011, Southgate et al., 2014). On average, infants contributed 5 fNIRS trials per condition to the analyses: 5 trials in the Native_FacialAction condition (range = 3–10) and 5 trials in the Foreign_FacialAction condition (range = 3–8). The number of included fNIRS trials did not differ between the two conditions (*p* = .83).

### EMG recording and processing

**Fig. 2:**
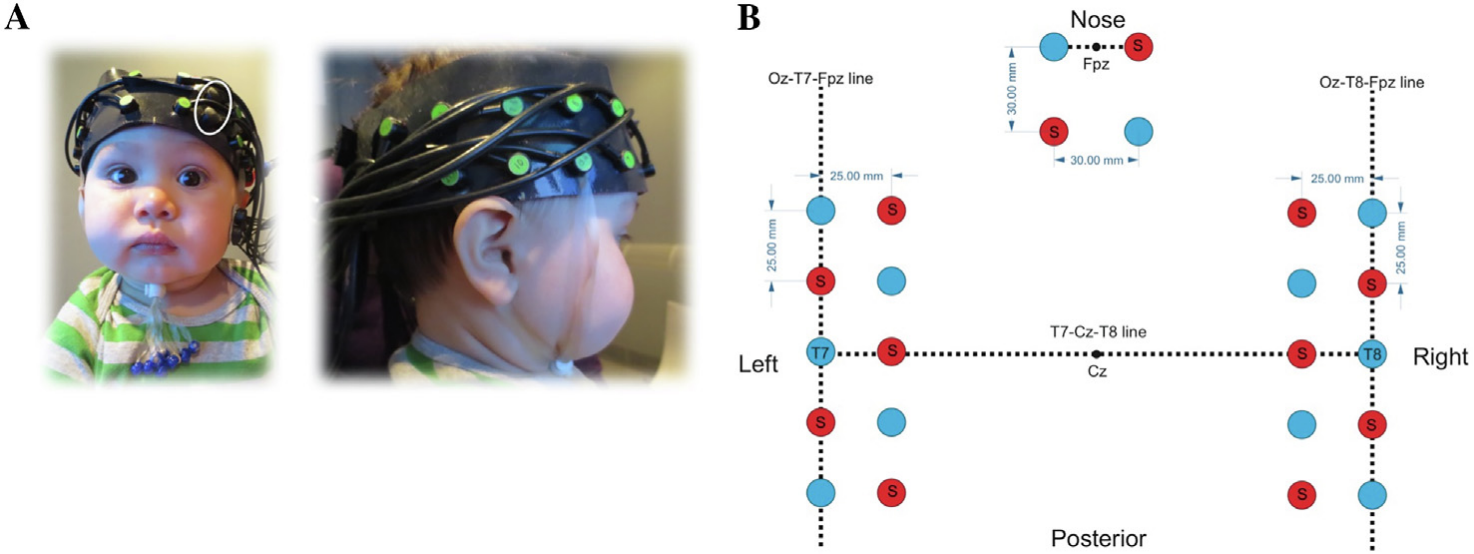
(A) Participant wearing the fNIRS headgear, showing the distribution of source and detector optodes over the temporal and frontal cortex. The location of the frontalis EMG electrodes is indicated with a white circle. (B) Representation of the location of the sources and detectors in reference to the 10–20 system.

Bipolar EMG recordings were made using pediatric surface Ag/AgCl electrodes that were placed on the cheek and forehead following recommendations by Fridlund and Cacioppo (1986) with an inter-electrode spacing of approximately 1 cm to measure activation over the masseter and frontalis muscle areas, respectively. The electrodes on the forehead were embedded within the fNIRS headgear, with the inferior electrode affixed approximately 1 cm above the upper border of the middle of the brow and the second electrode placed 1 cm superior to the first one (Fridlund & Cacioppo, 1986) (see Fig. 2A). A stretchy silicone band was tightly affixed over the headgear to ensure that the fNIRS optodes and EMG electrodes made contact with the scalp and the skin, respectively. The electrodes were connected to Myon wireless transmitter boxes that amplified the electrical muscle activation, which was in turn recorded using ProEMG at a sampling rate of 2000 Hz. After recording, the EMG signal was filtered (high-pass: 30 Hz; low-pass: 500 Hz), smoothed (root mean square over 20-ms bins), and rectified.

The EMG signal was segmented into 3000-ms epochs, and the average activity in each epoch was normalized (i.e., expressed as *z*-scores) within each participant and each muscle group (masseter and frontalis regions) before the epochs for each trial type were averaged together. Because facial mimicry can be defined as the presence of greater activation over corresponding muscles than over non-corresponding muscles during the observation of facial actions (e.g., McIntosh et al., 2006, Oberman et al., 2009), we calculated a mimicry score per trial by subtracting EMG activity over the non-corresponding muscle region from EMG activity over the corresponding muscle region (e.g., on an eyebrow trial, we subtracted activity over the masseter region from activity over the frontalis region so that a more positive score indicates more mimicry).

### fNIRS recording and processing

fNIRS data were recorded using the University College London (UCL)–NIRS topography system, which uses two continuous wavelengths of near-infrared light (770 and 850 nm) to detect changes in oxyhemoglobin (HbO_2_) and deoxyhemoglobin (HHb) concentrations in the brain and has a sampling rate of 10 Hz (Everdell et al., 2005). Infants wore custom-built headgear with a total of 26 channels, with a source–detector separation of 25 mm over the temporal areas and 4 channels with a source–detector separation of 30 mm over the frontal area. The headgear was placed so that the third optode was centered above the pre-auricular point (directly over T7 and T8 according to the 10–20 system) (see Fig. 2B). Based on the understanding of the transportation of near-infrared light through tissue, this source–detector separation was predicted to penetrate up to a depth of approximately 12.5 mm from the skin surface for the temporal areas and a depth of approximately 15 mm from the skin surface for the frontal areas, allowing measurement of both the gyri and parts of the sulci near the surface of the cortex (Lloyd-Fox, Blasi, & Elwell, 2010). Previous research using coregistration of fNIRS and MRI using the same array design for the temporal areas has demonstrated that this design permits measurement of brain responses in cortical regions corresponding to the inferior frontal gyrus (IFG), superior temporal sulcus (STS), and TPJ areas (see Lloyd-Fox et al., 2014). The frontal array was designed to measure brain responses in mPFC (Kida and Shinohara, 2013, Minagawa-Kawai et al., 2009).

Data were preprocessed using a MATLAB software package called HOMER2 (MGH–Martinos Center for Biomedical Imaging, Boston, MA, USA) and analyzed using a combination of custom MATLAB scripts and the Statistical Parametric Mapping (SPM)–NIRS toolbox (Ye, Tak, Jang, Jung, & Jang, 2009). Data were converted to .nirs format, and channels were excluded if the magnitude of the signal was greater than 97% or less than 3% of the total range for longer than 5 s during the recording. Channels with raw intensities smaller than 0.001 or larger than 10 were excluded, and motion artifacts were corrected using wavelet analyses with 0.5 times the interquartile range. Hereafter, the data were band-pass filtered (high-pass: 0.01 Hz; low-pass: 0.80 Hz) to attenuate slow drifts and high-frequency noise. The data were then converted to relative concentrations of HbO_2_ and HHb using the modified Beer–Lambert law. We excluded from analysis any channels that did not yield clean data for at least 70% of the infants. This resulted in the exclusion of three channels associated with a source that was faulty for a subset of the assessments (Channels 21, 23, and 24). As in previous infant fNIRS studies (e.g., Southgate et al., 2014), infants for whom more than 30% of remaining channels were excluded due to weak or noisy signal were excluded from analysis (*n* = 2). Note that for these 2 excluded infants, the intensities were very weak over more than 30% of the channels because the fNIRS headgear was not fitted properly.

Our data analysis approach was determined a priori and followed Southgate et al. (2014). For each infant, we constructed a design matrix with five regressors. The first regressor modeled the Native Reminder trials (duration = 6 s), the second regressor modeled the Foreign Reminder trials (duration = 6 s), the third regressor modeled the Native_FacialAction trials (duration = 10 s), the fourth regressor modeled the Foreign_FacialAction trials (duration = 10 s), and the fifth regressor modeled the Baseline trials (duration = 8 s). Excluded trial periods were set to zero, effectively removing them from the analyses. The regressors were convolved with the standard hemodynamic response function to make the design matrix (Friston, Ashburner, Kiebel, Nichols, & Penny, 2011). This design matrix was then fit to the data using the general linear model as implemented in the SPM–NIRS toolbox (Ye et al., 2009). Beta parameters were obtained for each infant for each of the regressors. The betas were then used to calculate a contrast between the different conditions of interest for each infant. Although in principle the HbO_2_ and HHb responses should be coupled, with HbO_2_ responses going up and HHb responses going down in response to stimulus presentation, studies with infant participants often do not find statistically significant HHb changes (Lloyd-Fox et al., 2010, Lloyd-Fox et al., 2015). Therefore, as in previous infant fNIRS studies, our analyses focused on changes in HbO_2_.

To ensure statistical reliability, and given that previous research has shown that our cortical areas of interest (e.g., STS, TPJ, IFG) are unlikely to span just one channel (Lloyd-Fox et al., 2014), we considered that activation at a single channel would be reliable only if it was accompanied by significant activation at an adjacent channel (Lloyd-Fox et al., 2011, Southgate et al., 2014). To implement this, we created a Monte Carlo simulation of *p* values at every channel of our particular array and, on 10,000 cycles, tested whether *p* values for two or more adjacent channels fell below a specific channel threshold. We repeated the simulations for a range of channel thresholds and then selected the channel threshold that led to a whole-array threshold of *p* < .05 for finding two adjacent channels activated by chance. The appropriate channel threshold was 0.0407, so we considered only effects present at *p *< .0407 in two adjacent channels to be interpretable.

## Results

### EMG

**Fig. 3:**
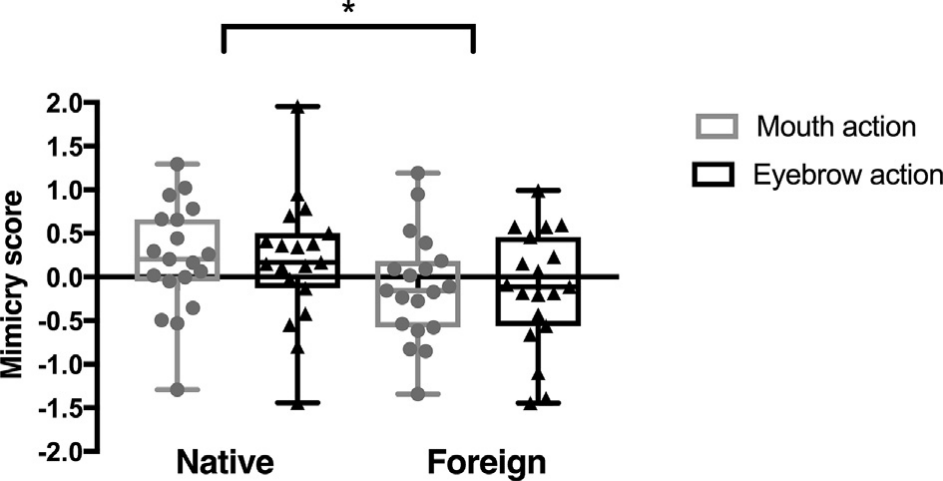
Box and whisker plots of the mimicry scores during the observation of eyebrow and mouth actions in the Native and Foreign mimicry conditions. The horizontal line within the box indicates the median, the boundaries of the box indicate the 25th and 75th percentiles, and the whiskers indicate the highest and lowest values. The circles and triangles represent the individual data points. ^*^p < .05.

A repeated-measures analysis on the mimicry scores (activation over the corresponding muscle region minus activation over the non-corresponding muscle region) with condition (Native vs. Foreign speaker) and action type (Eyebrow vs. Mouth) as within-participant factors demonstrated a significant main effect of condition, *F*(1, 18) = 6.07, *p* = .024, *η*_p_^2^ = .252. There were no other significant main effects or interactions. As can be seen in Fig. 3, infants showed significantly greater mimicry of facial actions performed by the native speaker compared to the foreign speaker. The mouth and eyebrow mimicry scores in the Native condition were not significantly different from zero (*p*s > .149), but the average mimicry score in the Native condition was, *t*(18) = 2.407, *p* = .027. This demonstrates that, overall, infants were significantly more likely to show greater activation over the corresponding facial muscles compared to the non-corresponding facial muscles when they observed the facial actions performed by the native speaker. (See the online supplementary material for the same analyses performed on the individual muscle activation as well as a depiction of the EMG signal over the masseter and frontalis muscle regions time-locked to the onset of the facial actions.)

#### Baseline correction

Note that the analyses reported above were planned a priori and followed our previous work (de Klerk et al., 2018). There are several reasons why we did not use baseline-corrected EMG values in these analyses. First, if we define mimicry as a relative pattern of muscle activation in which corresponding facial muscles are activated to a greater extent than non-corresponding facial muscles, it does not seem necessary to perform a baseline correction. Instead, by transforming the EMG activity to *z*-scores and calculating mimicry scores, we can measure infants’ tendency to selectively activate the corresponding facial muscles to a greater degree than the non-corresponding facial muscles during the observation of facial actions. The second reason why we did not subtract activity during the baseline period from activity during the trials was that one of the main reasons for excluding trials at this age were vocalizations, and infants tended to vocalize a lot during the baseline stimuli. To maximize the number of trials that we could include, and hence the number of infants that could be included in the analyses, we decided not to also require there to be a valid baseline preceding each trial. However, on request of the reviewers, we have recoded the videos of the sessions to identify all valid baselines and reanalyzed the data using baseline-corrected values. In these analyses, the effect of condition was no longer significant (*p* = .193) and there was no evidence for mimicry. (See the supplementary material for more details as well as potential explanations for this discrepancy in the findings.)

### fNIRS

**Fig. 4:**
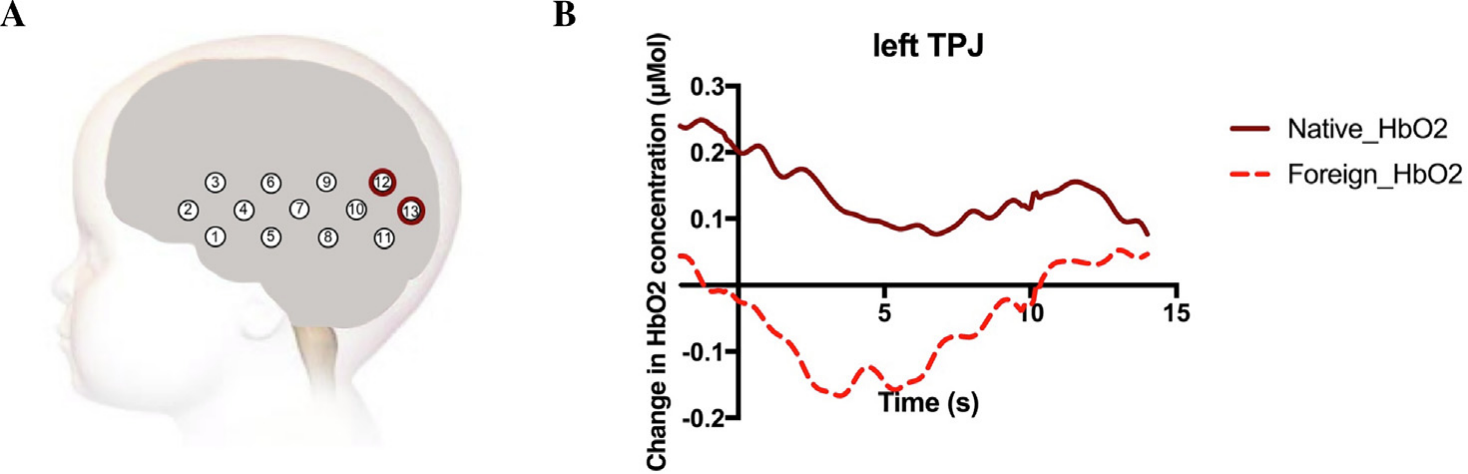
(A) The location of the fNIRS channels with a significantly greater hemodynamic response (based on HbO_2_) for the Native_FacialAction condition compared to the Foreign_FacialAction condition. (B) Time course of the grand averaged hemodynamic responses over the left temporal parietal cortex (Channels 12 and 13) for both conditions. Note that the data are not baseline corrected because the SPM–NIRS toolbox analyzes the entire fNIRS time series.

*t* Tests revealed that the left temporal parietal cortex was sensitive to the linguistic status of the models. This region showed a significantly greater hemodynamic response (based on HbO_2_) both when the Native_FacialAction condition was contrasted to the Baseline condition and when it was compared to the Foreign_FacialAction condition (see Table 1). This effect was present at p < .0407 over two adjacent channels (Channels 12 and 13; see Fig. 4). No channels showed a significantly greater response in the Foreign_FacialAction condition compared to the Native_FacialAction condition. Although we found one channel over the frontal cortex (mPFC area) that showed a significant hemodynamic response for the Foreign_FacialAction condition compared to the Baseline condition, there was no significant difference between the two conditions over this channel. (See Supplementary Fig. 2 for the beta values for all channels.)

**Table 1:** Channels that showed a significantly greater hemodynamic response for the contrasts of interest.

Channel	*t* Value	*p* Value
Native_FacialAction > Baseline		
12 (temporal parietal)	2.758	.013
13 (temporal parietal)	3.395	.003

Foreign_FacialAction > Baseline		
2 (inferior frontal)	2.081	.048
29 (frontal)	2.428	.023

Native_FacialAction > Foreign_FacialAction		
12 (temporal parietal)	2.957	.008
13 (temporal parietal)	2.243	.037

*Note.* The brain area that the channel is located over is indicated in parentheses.

### Relationship between EMG and fNIRS data

**Fig. 5:**
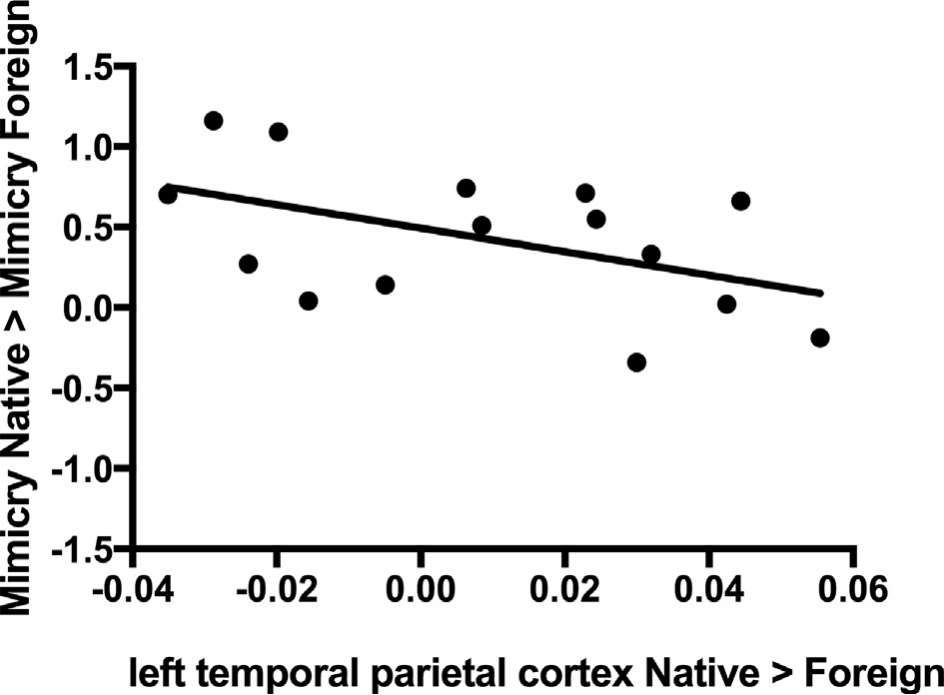
Relationship between the betas for the contrast Native_FacialAction > Foreign_FacialAction over the left temporal parietal cortex (Channels 12 and 13) and the mimicry difference score (mimicry in the Native condition minus mimicry in the Foreign condition), *r*(13) =  − .487, *p* = .066.

We initially planned to perform correlational analyses to investigate the relationship between the EMG and fNIRS results. These analyses are underpowered (*N* = 15)^3^ due to the relatively high dropout rate, however, we report the results here for completeness. We found a marginally significant negative correlation between the Native_FacialAction > Foreign_FacialAction contrast over the left temporal parietal cortex (Channels 12 and 13) and the differential mimicry response (Native_FacialAction − Foreign_FacialAction), *r*(13) = −.487, *p* = .066 (see Fig. 5). Suggesting that infants who showed a greater hemodynamic response over the left temporoparietal area when observing facial actions performed by the native speaker compared with the foreign speaker showed less mimicry of facial actions performed by the native speaker compared with the foreign speaker (see Fig. 5). Note that because this correlational analysis is underpowered, it needs to be interpreted with caution.

### Choice task

Infants were significantly more likely to choose a picture of the native speaker compared to the foreign speaker, *χ*^2^(1) = 6.54, *p* = .011. Of the 27 infants included in the EMG and/or fNIRS analyses, 17 chose the native speaker, 5 chose the foreign speaker, and 5 did not choose.

## Discussion

This study is the first to demonstrate that the linguistic status of an observed model modulates facial mimicry in infancy and that this modulation is accompanied by changes in neural activation over the left temporal parietal cortex. These results show that one of the hallmarks of mimicry—that it is modulated by cues to group membership—seems to be present from at least 11 months of age.

What is the psychological mechanism underlying infants’ tendency to selectively mimic the native speaker? It is important to note from the outset that the modulation of mimicry by the language of the model does not imply that infants conceived of the native speaker as a member of their in-group (Liberman et al., 2017). Infants are clearly sensitive to cues that correlate with group membership, such as language familiarity, and this sensitivity modulates their attention and expectations (Begus, Gliga, & Southgate, 2016). For example, infants may have considered the native speaker to be a more useful source of information (Begus et al., 2016) or a more competent person (Brooker & Poulin-Dubois, 2013) because she correctly labeled the familiar objects, whereas they could not understand the foreign speaker. As a result of this, the native speaker may have captured the infants’ attention to a greater extent, leading to increased encoding of her facial actions and, consequently, greater activation of the associated motor representations and greater mimicry—a process called *input modulation* (Heyes, 2013). Although the fact that we did not find any significant differences in the number of included trials between the conditions seems to speak against this interpretation, looking is not necessarily equivalent to attending (Aslin, 2012); therefore, we cannot completely rule out the possibility that there may have been enhanced encoding of the facial actions performed by the native speaker.

It is also possible that, like adults, infants may have had a greater motivation to affiliate with the native speaker, and mimicking her facial actions could have functioned as a means to communicate their similarity to the model (van Baaren et al., 2009). Indeed, infants’ preference for the native speaker in the choice test seems consistent with the idea that infants have an early emerging preference to interact with familiar others (Kinzler et al., 2007).

Although the facial mimicry we observed was subtle and mainly detectable by EMG, previous studies suggest that our emotions and social perceptions can be influenced by facial stimuli that we cannot consciously perceive (Bornstein et al., 1987, Dimberg et al., 2000, Li et al., 2008, Svetieva, 2014, Sweeny et al., 2009). Therefore, regardless of the underlying mechanism—attentional effects or affiliative motivations—the increased mimicry of in-group members during infancy is likely to have a positive influence on social affiliation (Kavanagh and Winkielman, 2016, van Baaren et al., 2009) and, therefore, can be expected to be reinforced over the course of development (Heyes, 2017).

A question that remains is how the facial mimicry that we measured in the current study relates to the overt mimicry behaviours observed in the original adult studies on behavioral mimicry (e.g., Chartrand & Bargh, 1999). In the adult literature, facial mimicry is generally placed in the same context as the spontaneous mimicry of other nonverbal behaviors such as postures and gestures (e.g., Chartrand and Lakin, 2013, Stel and Vonk, 2010, van Baaren et al., 2009). Indeed, facial mimicry as measured by EMG and mimicry of postures, gestures, and mannerisms share many properties; they both seem to occur without conscious awareness (Chartrand and Bargh, 1999, Dimberg et al., 2000), are influenced by the same factors such as group membership (Bourgeois and Hess, 2008, Yabar et al., 2006), and are supported by similar neural mechanisms (Likowski et al., 2012, Wang and Hamilton, 2012). Furthermore, it has been suggested that the subtle mimicry that can be measured by EMG may be a building block for more overt and extended matching (Moody and McIntosh, 2006, Moody and McIntosh, 2011). Potentially, this constitutes a quantitative change rather than a qualitative change, where the mimicry becomes overtly visible whenever the activation of the motor representation in the mimicker reaches a certain threshold. Future research will need to investigate the relationship between overt behavioral mimicry and subthreshold mimicry measured by EMG in more detail to determine whether they are indeed two sides of the same coin or rather distinct processes.

The fact that EMG can pick up on relatively subtle mimicry may also explain the discrepancy between the current study’s findings and those of van Schaik and Hunnius (2016). In that study, 4-year-olds, but not 3-year-olds, showed modulation of behavioral mimicry (e.g., yawning, rubbing the lips) depending on whether the model was wearing the same t-shirt color as them. One possibility is that children younger than 4 years are not sensitive to superficial attributes such as t-shirt color (van Schaik & Hunnius, 2016) or that they lack experience with being divided into teams based on such attributes. Alternatively, it could be that coding of overt behavioral mimicry is less sensitive than facial mimicry as measured by EMG. Future research should investigate whether a minimal group paradigm might elicit selective mimicry in children younger than 4 years when more sensitive measures, such as facial mimicry as measured by EMG, are used. Such an approach would also allow researchers to investigate whether the selective mimicry effects found in the current study would hold when language, familiarity, and competence factors are controlled for across the in- and out-group members.

It should be noted that although we found evidence for mimicry overall, the mimicry scores for the mouth and eyebrow actions separately were not significantly different from zero. In addition, when we performed baseline-corrected analyses (see supplementary material), the effect of condition became nonsignificant and there was no evidence for mimicry, suggesting that the mimicry responses we found here might not be as robust as those reported in previous studies (e.g., Datyner, Henry, & Richmond, 2017; Geangu, Quadrelli, Conte, Croci, & Turati, 2016; Isomura & Nakano, 2016). There are several possible explanations for this. First of all, most of the previous developmental studies on facial mimicry used emotional facial expressions as the stimuli. One possibility is that muscle responses to these stimuli reflect emotional contagion, a process in which the observed stimuli induce a corresponding emotional state in the child, resulting in the activation of corresponding facial muscles, whereas mimicry of nonemotional facial expressions, such as those used in the current study, reflects subtler motor mimicry (Hess and Fischer, 2014, Moody and McIntosh, 2011). Future research will need to investigate whether there are differences in the mechanisms underlying the mimicry of emotional and nonemotional facial actions.

A recent study suggests that the development of facial mimicry is supported by parental imitation (de Klerk, Lamy-Yang, & Southgate, 2018). Thus, a second possibility is that the current study included a mixture of infants who receive high and low levels of maternal imitation, resulting in a relatively high level of variability in the mimicry responses, with some infants potentially not having received a sufficient amount of correlated sensorimotor experience to support the mimicry of the observed facial actions.

We found a greater hemodynamic response in channels overlying the left temporal parietal cortex during the observation of facial actions performed by the native speaker compared to the foreign speaker. The temporal parietal region, and the TPJ in particular, has been suggested to play a critical role in disambiguating signals arising from one’s own and others’ actions (Blakemore & Frith, 2003), and some evidence suggests that it may play a role in these processes from an early age (Filippetti, Lloyd-Fox, Longo, Farroni, & Johnson, 2015). Although the left lateralization of our results seems inconsistent with previous research mainly implicating the *right* TPJ in separating self- and other-generated signals (e.g., Spengler et al., 2009), a recent study suggests that the bilateral TPJ is involved in this process (Santiesteban, Banissy, Catmur, & Bird, 2015). One interpretation of our fNIRS findings is that the Native speaker condition may have posed higher demands on differentiating between self- and other-generated actions because it presented infants with a highly affiliative context where the urge to mimic was strong (for similar results with adult participants, see Rauchbauer et al., 2015). In other words, a possible consequence of infants’ enhanced tendency to mimic the native speaker may have been an increased self–other blurring, which led to greater compensatory activity over the temporal parietal cortex. In line with this interpretation, the marginally significant negative correlation between the betas for the contrast Native_FacialAction > Foreign_FacialAction over the left temporal parietal cortex and the mimicry difference score suggests that those infants who showed a greater amount of temporal parietal cortex activation may have maintained greater self–other differentiation and showed a less pronounced selective mimicry response. However, given that this correlational analysis was heavily underpowered, this finding needs to be replicated in a larger sample. In addition, considering the limited amount of research on the role of the TPJ in self–other differentiation during infancy, future research will need to further investigate the role of this area in inhibiting mimicry responses during infancy. Finally, the spatial resolution of fNIRS does not allow us to say with certainty that the significant channels did not, at least in part, overlie the posterior STS (pSTS). Therefore, another possible interpretation of our fNIRS findings is that greater activation over the temporal parietal cortex during the observation of facial actions performed by the native speaker reflects input modulation, that is, the Native speaker condition may have captured the infants’ attention to a greater extent, leading to enhanced encoding of her facial actions as indicated by greater activation over the pSTS. This interpretation would be consistent with a previous study in which we found greater activation over the pSTS in the condition associated with greater facial mimicry in 4-month-old infants (de Klerk et al., 2018). However, given that the significant channels in the current study are located in a more posterior position on the scalp, they are unlikely to reflect activation of the exact same area. Future fNIRS–fMRI coregistration work would be beneficial to help tease apart the role of adjacent cortical areas in the modulation of mimicry responses.

One potential concern may be that the differences in facial mimicry between the two conditions led to subtle artifacts in the fNIRS data that created the differences in the hemodynamic response over the left temporal parietal area. We should note that it seems unlikely that facial muscle contractions that cannot be seen with the naked eye would cause artifacts large enough to result in a significant difference between the two conditions. In addition, even if this did happen, it seems highly unlikely that this would have specifically affected two adjacent channels over the left hemisphere over a cortical area that is the farthest removed from the facial muscles rather than the frontal channels that are directly on the forehead.

Unlike in adults (Wang et al., 2011), we did not find involvement of the mPFC in the modulation of mimicry in infants. One possibility is that our frontal array was not optimal for measuring responses in the mPFC, although previous studies using similar array designs have reported differential responses over this area (Kida and Shinohara, 2013, Minagawa-Kawai et al., 2009). Another possibility is that at this relatively young age, the selective mimicry responses were mainly driven by bottom-up attentional processes, such as the tendency to pay more attention to familiar others, whereas more top-down mechanisms start to play a role in modulating mimicry behavior only once myelination of the relevant long-range connections with the mPFC is more established (Johnson, Grossmann, & Kadosh, 2009). Future research measuring functional connectivity during mimicry behaviors over the course of development will need to investigate this further.

Taken together, our results demonstrate that facial mimicry is flexibly modulated by cues to group membership from at least 11 months of age. Although the exact mechanisms underlying this selective mimicry response will need to be investigated in future research, these findings suggest that the foundations for the role that mimicry plays in facilitating social bonds seem to be present during the first year of life.

## Footnotes

1 Note that because the mother was on maternity leave at the time of the experiment, this infant was not exposed to English regularly. In addition, for this session the second author was the main experimenter, and she spoke Italian to the mother and baby. The results did not change when we excluded this infant.

2 Note that we did not split the fNIRS trials into four categories because not all infants had at least 3 good 10-s trials for the four different trial types. Instead, we collapsed the Mouth and Eyebrow trials into the Native_FacialAction and Foreign_FacialAction conditions.

3 Of the 17 infants who provided both good EMG and good NIRS data, 2 did not have data over Channels 12 and 13.
