# Supplementary material for: Selective facial mimicry of native over foreign speakers in preverbal infants
Source: J Exp Child Psychol. 2019 Jul;183:33–47. doi: 10.1016/j.jecp.2019.01.015 (PMC6478146; doi:10.1016/j.jecp.2019.01.015)
Supplement: Supplementary data 1 [file mmc1.docx]

**Supplementary Materials**

**S1. Baseline correction**

Like in our previous work (de Klerk et al., 2018), we did not perform a baseline-correction on the data for our main analyses. Here we report reviewer-requested post-hoc analyses performed on baseline-corrected data. The baseline period was taken from 1,250 to 250 ms preceding the onset of the trial, based on previous infant facial EMG work by Isomura and Nakano (2016). For those trials that were not preceded by a valid baseline (e.g. because the infant was vocalizing or had something in their mouth), we used the average of all valid baseline periods. Using these baseline-corrected data, we found that the effect of condition was no longer significant, *F* (1, 17) = 1.839, *p* = .193, *η_p_^2^* = .098, and that there was no evidence for mimicry, all *p*’s > .270. There are several potential explanations for this. First of all, it is likely that there was a lot of variability in the subthreshold facial muscle responses during the baseline stimuli, and therefore the fact that for some infants the baseline activation was only based on a handful of valid baseline trials may have had a big impact on the results. E.g. if the infant by chance showed a strong masseter response during only three valid baseline trials, and these three trials were averaged together and subtracted from the activation during all the trials that were not preceded by a valid baseline, then this would have created a suboptimal estimate of the amount of EMG activity during these trials. Additionally, as the baseline durations were fixed at 8 seconds, it is possible that some infants started to anticipate the onset of the next facial action trial, potentially resulting in unspecific anticipatory facial EMG activity during the baseline period. Therefore, we believe that using the z-scored EMG activity instead of the baseline-corrected EMG activity is the more appropriate approach for our dataset.

**S.2. Supplementary EMG analyses**

In the paper we reported analyses performed on the Mimicry scores. In these reviewer-requested post-hoc analyses we demonstrate that the results are the same when we analyse the individual muscle activations instead. For this analysis we calculated the mean EMG-activity (z-scores) over the frontalis and masseter region during the observation of eyebrow and mouth actions performed by the native and foreign speaker for each 500 ms time-window after stimulus onset. As expected based on the averaged responses reported in the main manuscript, a repeated measures ANOVA with timebin (six 500 ms timebins), condition (Native vs. Foreign), action (Mouth vs. Eyebrow), and muscle (Masseter vs. Frontalis), demonstrated a significant interaction between condition, action, and muscle, *F(*1,18)=5.803, *p* = 027. There was a significant interaction between action and muscle in the Native condition only, *F*(1,18)=4.91, *p*=.040, again demonstrating that infants activate their facial muscles differentially depending on whether they observe the native speaker perform mouth or eyebrow actions. However, consistent with the absence of significant mimicry when we consider the eyebrow and mouth action condition separately as was reported in the main manuscript, the main effects of muscle for the Mouth or Eyebrow actions were not significant, *p*’s >.163.

**
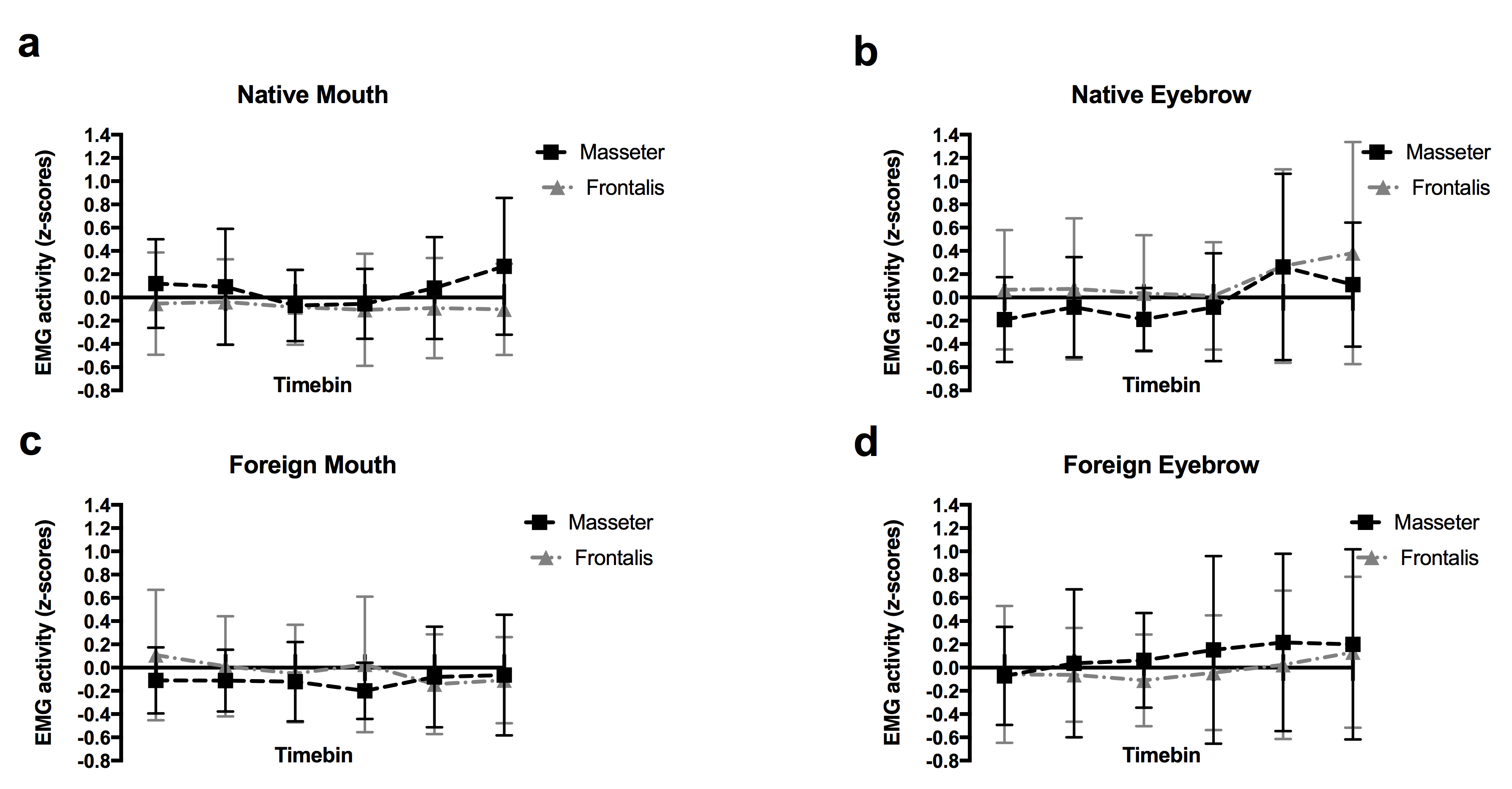
***Supplementary Figure 1.* Mean EMG-activity (z-scores) over the frontalis and masseter region during the observation of eyebrow and mouth actions performed by the native and foreign speaker for each 500 ms time-window after stimulus onset. Error bars represent the standard deviation.

**
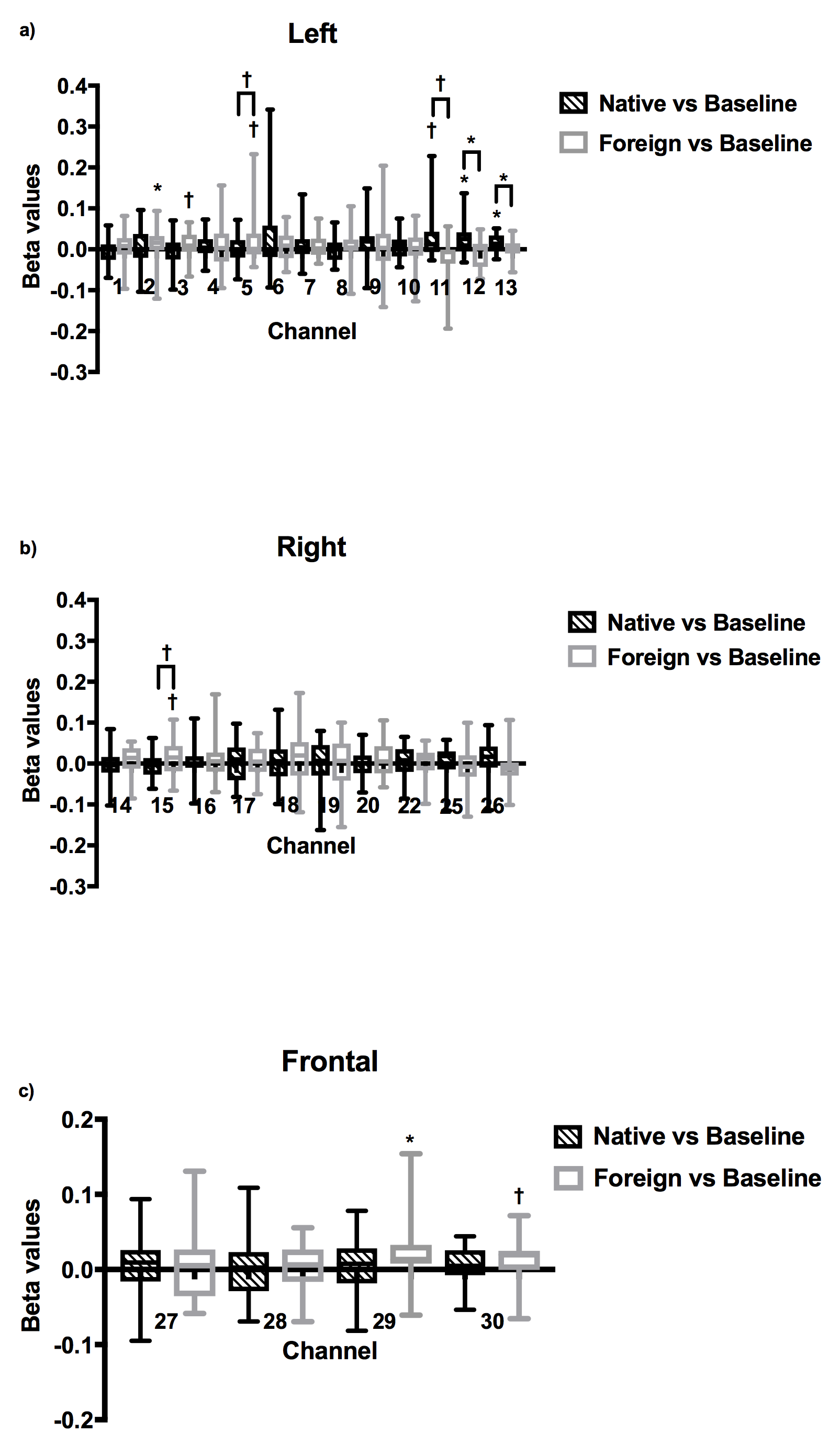
**

*Supplementary Figure 2.* Box and whisker plots of the mean beta values over all the included channels for the Native vs. Baseline and Foreign vs. Baseline contrast. The horizontal line within the box indicates the median, the boundaries of the box indicate the 25^th^ and 75^th^ percentile, and the whiskers indicate the highest and lowest values. * *p* < .05, † .05 < *p* < .1.
